# Genomic characterization of Listeria monocytogenes isolated from normally sterile human body fluids in Lithuania from 2016 to 2021

**DOI:** 10.1099/mgen.0.001410

**Published:** 2025-05-20

**Authors:** Anželika Slavinska, Elita Jauneikaite, Ugnė Meškytė, Agnė Kirkliauskienė, Adam Misevič, Aurelija Petrutienė, Nomeda Kuisiene

**Affiliations:** 1 Department of Microbiology and Biotechnology, Institute of Biosciences of Vilnius University Life Sciences Centre, 10257 Vilnius, Lithuania; 2 NIHR Health Protection Research Unit in Healthcare Associated Infections and Antimicrobial Resistance, Imperial College London, London, UK; 3 Faculty of Medicine, Institute of Biomedical science, Vilnius University, 03101 Vilnius, Lithuania; 4 Department of Clinical Investigations of the National Public Health Surveillance Laboratory, 10210 Vilnius, Lithuania

**Keywords:** antimicrobial resistance, genotyping, *Listeria monocytogenes*, pathogenicity, virulence, whole-genome sequencing

## Abstract

*Listeria monocytogenes* is a saprophytic gram-positive bacterium and opportunistic foodborne pathogen that can cause listeriosis in humans. The incidence of listeriosis has been rising globally and, despite antimicrobial treatment, the mortality rates associated with the most severe forms of listeriosis such as sepsis, meningitis and meningoencephalitis remain high. The notification of listeriosis in humans is mandatory in Lithuania, and up to 20 cases are reported annually. However, no studies have described the detailed virulence and antimicrobial susceptibility profiles of any clinical *L. monocytogenes* strains in Lithuania. Accordingly, this study aimed to describe the antibiotic susceptibility of invasive *L. monocytogenes* and perform in-depth characterization of strains isolated from patients with neuroinfections through whole-genome sequencing. A total of 70 isolates were collected, mostly from infected patients aged 65 or older, between 2016 and 2021 : 41 (58.6%) from blood, 19 (27.1%) from cerebrospinal fluid, 5 (7.1%) from wounds, 1 (1.4%) from pleural fluid and 1 (1.4%) from a brain abscess. Two phylogenetic lineages were identified—I (*n* = 16/70, 22.9%) and II (*n* = 54/70, 77.1%)—along with three serogroups—IIa (*n* = 53/70, 75.7%), IVb (*n* = 16/70, 22.9%), and IIc (*n* = 1/70, 1.4%). Genomic analysis of 20 isolates showed a high level of diversity with seven genotypes: ST6 (*n* = 6), ST155 (*n* = 5), ST8 (*n* = 4), ST504 (*n* = 2) and singletons for ST37, ST451 and ST2. Phylogenetic analysis clustered these isolates into two clades defined by serogroups IVb and IIa. Notably, five isolates were clustered tightly together (difference of 6–48 core SNPs from reference and 0, 4 or 44 SNPs from each other) with ST155, previously reported in a European outbreak. Comparison with publicly available *L. monocytogenes* genomes did not identify unique clusters or genotypes. No acquired antimicrobial resistance genes were identified. Our study highlights the complementary value of whole-genome sequencing in routine PCR-based surveillance in Lithuania. This is the first study to characterize and compare genomes for *L. monocytogenes* associated with neuroinfections in Lithuania using whole-genome sequencing. The retrospective detection of the ST155 clone underscores the need for a review and strengthening of epidemiological surveillance strategies in clinical and non-clinical settings in Lithuania.

**Impact Statement**

This study employed whole-genome sequencing (WGS) to provide the first comprehensive molecular and phylogenetic characterization of clinical *L. monocytogenes* isolates from Lithuania. The phenotypic analysis of antimicrobial susceptibility demonstrated that all isolates were susceptible to clinically relevant antibiotics, and no acquired antimicrobial resistance genes were detected using WGS. The isolates showed high genetic diversity across two phylogenetic lineages, and five were closely related to ST155, a sequence type genetically related to the Omikron1 clone, which was linked to listeriosis outbreaks in five European countries through its contamination of ready-to-eat fish products. Our findings highlight the critical need for reviewing and enhancing epidemiological surveillance strategies in Lithuania. This research contributes valuable WGS data from Lithuania to enrich international databases, aiding global epidemiological surveillance of *L. monocytogenes* and improving our understanding of its transmission and public health risks.

## Data Summary

The raw sequencing reads from the *L. monocytogenes* isolates generated in this study are available from the European Nucleotide Archive (https://www.ebi.ac.uk/ena/browser/home) under project accession number PRJEB80562; accession numbers for individual isolates are provided in Table S1. Draft genomes of the *L. monocytogenes* strains were submitted to the BIGSdb-*Lm* database (https://bigsdb.pasteur.fr/listeria/) under accession numbers 110263–110281 and 108726.

## Introduction

*Listeria monocytogenes* causes listeriosis, a rare but severe infection that can present as non-invasive gastroenteritis or invasive forms such as septicemia, maternal-neonatal infections, meningitis and encephalitis [1]. With pregnant women, infants, immunocompromised individuals and older adults demonstrating the worst outcomes [2]. Listeriosis is usually acquired via consumption of contaminated food [3] but is also widely present in nature in a range of animals, plants and environments [4]. Globally, 0.1 to 10 cases per 1 million population of listeriosis are reported annually [5]. In 2023, a total of 2952 invasive *Listeria monocytogenes* cases were confirmed across 27 EU Member States, resulting in a notification rate of 0.66 per 100,000 population—the highest recorded since 2007. A statistically significant upward trend was observed from 2019 to 2023 [6]. Although the numbers of listeriosis cases are low, the infections have a high hospitalization rate [6,7], adverse outcomes and mortality [8,9]. Listeriosis has the highest case fatality rate among zoonotic diseases, reaching 19.7% [6], and is among the leading causes of death from foodborne infections in high-income countries [7]. Additionally, its potential to cause foodborne outbreaks [10,12] makes * L. monocytogenes* an important public health threat.

The β-lactam antibiotics penicillin and aminopenicillins, including ampicillin and amoxicillin, are the first-line treatments for listeriosis [13,15]. β-lactam antibiotics are usually combined with an aminoglycoside, most commonly gentamicin [13,14, 16]. Trimethoprim/sulfamethoxazole is used in cases of suspected penicillin allergy [16]. The use of fluoroquinolones, rifampicin, linezolid and vancomycin has also been reported [17]. Unlike many other important human pathogens, *L. monocytogenes* has largely retained its susceptibility to antibiotics that have been used for decades in both humans and animals, such as penicillins [18]. *L. monocytogenes* is naturally resistant to specific antimicrobials, including third-generation cephalosporins and monobactams, because it does not have the appropriate penicillin-binding proteins [14] and is inherently resistant to first-generation quinolones, fosfomycin, cationic antimicrobial peptides, lincomycin and sulfonamides due to intrinsic resistance genes, such as *norB* [19], *fosX* [20,21], *mprF* [22], *lin* [23] and *sul* [18]. The regulation of intrinsic resistance genes in *L. monocytogenes* is highly complex, making it difficult to predict phenotypes [24]. In contrast, acquired antimicrobial resistance in *L. monocytogenes* can be accurately predicted from genomic data, as demonstrated in recent studies [18].

Molecular and genomics approaches have been used to investigate potential outbreaks and provide genotyping data on * L. monocytogenes* populations. *L. monocytogenes* has broadly been grouped into lineages I–IV [25]. It is known that isolates from humans and food sources predominantly fall into two phylogenetic lineages [26] consisting of serotypes 1/2a, 1/2b and 4b, which are responsible for the majority of human listeriosis cases; serotype 4b has also been assigned to the leading hypervirulent clones of clonal complexes (CCs) 1, 2, 4 and 6 [2,27,29]. Whole-genome sequencing (WGS) has become a widely used method for in-depth genotyping *L. monocytogenes* [30] and has proven useful in investigating the source of the outbreaks [31,32]; however, this method is rarely adopted for monitoring and routine surveillance, especially in countries with limited funding for epidemiological surveillance, such as Lithuania, where the notification of listeriosis in humans is mandatory and up to 20 cases of invasive listeriosis are reported annually (0.21–0.70 per 100 000 populations between 2019 and 2023) [6].

Notably, there have been no detailed studies on the virulence and antimicrobial susceptibility profiles of clinical *L. monocytogenes* strains circulating in Lithuania. Hence, this study assessed the antimicrobial susceptibility of clinical *L. monocytogenes* strains in Lithuania and characterized in detail isolates from neuroinfection cases using WGS.

## Methods

### Bacterial isolates

We examined 70 *L*. *monocytogenes* isolates provided by the National Public Health Surveillance Laboratory (Vilnius, Lithuania). The isolates were collected from different clinical samples between 10 October 2016 and 20 September 2021 (Table S1, available in the online version of this article). Pure *L. monocytogenes* cultures were stored at −80 °C in brain heart infusion broth (Liofilchem, Roseto degli Abruzzi, Italy) containing 20% glycerol. To recover the isolates from long-term storage, 10 µl of stock was cultured on sheep blood agar plates (bioMérieux, Marcy-l'Étoile, France) at 37 °C for 24–48 h. A single colony from a visually pure culture of each *L. monocytogenes* isolate was selected for analysis.

### Antibiotic susceptibility testing

Minimum inhibitory concentrations (MICs) for penicillin G (0.063–4.0 mg l^−1^), ampicillin (0.25–16.0 mg l^−1^), erythromycin (0.125–4.0 mg l^−1^), meropenem (0.25–16.0 mg l^−1^), tetracycline (0.5–4.0 mg l^−1^) and vancomycin (1.0–8.0 mg l^−1^) were determined using the MICRONAUT-S (Sifin diagnostics Gmbh, Berlin, Germany) broth microdilution method and interpreted according to the European Committee on Antimicrobial Susceptibility Testing (EUCAST) breakpoints v14.0 [33]. The EUCAST provides MIC breakpoint values for *L. monocytogenes* only for benzylpenicillin, ampicillin, meropenem and erythromycin. For other antibiotics, MIC values were determined based on EUCAST guidance for cases where species-specific breakpoints are not available. This approach considers MIC distributions, PK/PD data and available references to support the interpretation of susceptibility [34]. Antimicrobial susceptibility testing was performed using MICRONAUT-H Medium for fastidious bacteria (Sifin diagnostics Gmbh) following incubation at 35–37 °C in 5% CO_2_ for 22–24 h. For quality control, *Streptococcus pneumoniae* ATCC 49619 was used.

### Genomic DNA extraction

Genomic DNA was extracted from single bacterial colonies using a GeneJET Genomic DNA Purification Kit (Thermo Fisher Scientific, Waltham, MA, USA), following the manufacturer’s protocol. DNA quality was assessed by measuring absorbance at 260/280 nm with a BioPhotometer (Eppendorf, Hamburg, Germany), and DNA integrity was verified using 1% agarose gel electrophoresis.

### PCR characterization of isolates

Genes specific for the genus *Listeria* (*prs*), species *monocytogenes* (*isp*), lineages LI and LII (*L1*, *L2*) and serotypes (ORF2819, ORF2110, *lmo0737*, *lmo1118*) of *L. monocytogenes* were targeted using PCR to confirm the presence of *L. monocytogenes* and define the specific lineages as previously described [35,36]. Amplification products were visualized on 1% agarose gel.

### Whole-genome sequencing and bioinformatics analyses

Genomic DNA for 20 *L*. *monocytogenes* isolates from cerebrospinal fluid samples was sent to MicrobesNG for whole-genome sequencing (https://microbesng.com/). DNA libraries were prepared using a Nextera XT DNA Library Preparation Kit (Illumina, San Diego, CA, USA) following the manufacturer’s protocol and sequenced on a NovaSeq 6000 system (Illumina) using a 250 bp paired-end protocol.

#### Quality control

The quality of the raw reads was assessed using FastQC v0.12.1 (https://github.com/s-andrews/FastQC) and MultiQC v1.21 (https://github.com/MultiQC/MultiQC). Trimmomatic v0.39 (https://github.com/usadellab/Trimmomatic) [37] was used to trim low quality bases using the following parameters: LEADING: 20, TRAILING: 20, SLIDINGWINDOW: 5 : 30, MINLEN: 50. Bacterial species were confirmed using Kraken2 v2.1.3 (https://github.com/DerrickWood/kraken2) [38] and Bracken v2.9 (https://github.com/jenniferlu717/Bracken) [39] with the Kraken database (last updated 11 November 2023). Reads were *de novo* assembled using Spades v3.15.5 (https://github.com/ablab/spades) [40]. Draft genomes were assessed using Quast v5.2.0 (https://github.com/ablab/quast) [41] and CheckM2 v1.0.2 (https://github.com/chklovski/CheckM2) [42]. Bioawk v1.0 (https://github.com/lh3/bioawk) was used to remove contigs of ≤200 bp. A summary of the assembly statistics is provided in Table S2.

#### Characterization

Abricate v1.0.0 (https://github.com/tseemann/abricate) was used to detect acquired antimicrobial resistance genes using the Resfinder database (last updated 18 January 2023) [43]. The *Listeria monocytogenes* MLST, cgMLST and other database hosted by the Institut Pasteur (BIGSdb-*Lm*) (https://bigsdb.pasteur.fr/listeria/) [44] was used to detect intrinsic antimicrobial resistance genes. Genotypes were assigned using mlst v2.23.0 (https://github.com/tseemann/mlst) and BIGSdb-*Lm* [44]. MOB-suite v3.1.8 (https://github.com/phac-nml/mob-suite) [45] was used to reconstruct and characterize plasmids from draft assemblies. Virulence factor profiles were determined using the Virulence Factor Database [46] and BIGSdb-*Lm* [44]. PHAge Search Tool Enhanced Release (PHASTEST) [47] was used to assess the presence of prophage sequences. Incomplete prophages were excluded from analysis. The coding sequences and non-coding RNAs were predicted using Bakta v1.9.4 (https://github.com/oschwengers/bakta) [48]. The contigs were also annotated using the RAST server [49] to categorize genes into functional subsystems.

#### Phylogenetic analysis

Snippy v4.6.0 (https://github.com/tseemann/snippy) was used to call SNPs from the whole genome sequences of 20 *L*. *monocytogenes* isolates using closed reference genome EGDe (accession number: AL591824) and for a more detailed look into ST155 clones, we have used the recent ST155 genome (accession number: ERR7113321 [50], PubMLST assembly ID: 103485_20_06303) as reference. Recombination analysis was done using Gubbins v3.3.5 [51]. Recombination-free core SNPs were used to build a phylogenetic tree using IQ-TREE v2.0.3 [52] with the ‘GTR+G+ASC’ model, which was visualized and annotated using iTOL v6.9.1 (https://itol.embl.de/about.cgi) [53].

#### Comparative analysis

We compared our WGS results to 317 assembled genomes, downloaded from BIGSdb-*Lm* (https://bigsdb.pasteur.fr/listeria/), of isolates collected internationally (Table S3). A phylogenetic tree was constructed as described in the phylogenetic analysis. We compared the virulence profiles of *L. monocytogenes* isolates from Lithuania with those for a subset of 22 assembled genomes. The subset consisted only of genomes that had ≤30 allelic differences (and <300 contigs) with Lithuanian strains selected from a minimum spanning tree (MST) created using the GrapeTree tool and BIGSdb-*Lm* (https://bigsdb.pasteur.fr/listeria/) (Fig. S1). The GrapeTree was constructed based on the cgMLST1748 scheme and was colour-coded according to the country of isolation [44].

## Results

### Overview of clinical *L. monocytogenes* strains: typing and antimicrobial susceptibility

A total of 70 *L*. *monocytogenes* isolates collected between 2016 and 2021 were analysed in this study. Among them, 41 (58.6%) were isolated from blood, 19 (27.1%) from cerebrospinal fluid, 5 (7.1%) from wounds, 1 (1.4%) from pleural fluid and 1 (1.4%) from a brain abscess (Table S1). The highest number of isolates was recorded in 2020 (*n* = 23, 32.9%). The majority of isolates were from patients aged 65 years and older (*n* = 35, 50%), followed by the 45–64 (*n* = 19, 27.1%) and 25–44 age groups (*n* = 6, 8.6%); there were only 3 (4.3%) isolates from patients younger than 1 year. Single cases were identified in the 1–4 and 15–24 age groups (1.4% each). The ages of five patients were unknown.

All isolates (*n* = 70) expressed the *prs* and *isp* genes, confirming their identity as *L. monocytogenes*. We identified two phylogenetic lineages—I (*n* = 16/70, 22.9%) and II (*n* = 54/70, 77.1%)—and three serogroups—IIa (*n* = 53/70, 75.7%), IVb (*n* = 16/70, 22.9%) and IIc (*n* = 1/70, 1.4%) (Table S1). PCR serogroup IIa was found in isolates for 2016–2021, including all eight isolates in the 2021 collection (Table S1, Fig. S2A).

There was no significant difference in PCR serogroup distributions between different age groups. PCR serogroup IIa was present in all age groups and was the only PCR serogroup in patients aged less than 1 to 24 years. The highest PCR serogroup diversity was observed in the 45–64 age group (*n* = 19), where PCR serogroup IIa accounted for 11 cases (57.9%), PCR serogroup IVb for seven cases (36.8%) and PCR serogroup IIc for one case (5.3%). The largest number of cases was reported in the ≥65 years group (*n* = 35), with 29 cases (82.9%) associated with PCR serogroup IIa and six (17.1%) with PCR serogroup IVb (Fig. S2B).

All *L. monocytogenes* isolates were susceptible to penicillin G, ampicillin and erythromycin. 32 (45.7%) *L*. *monocytogenes* isolates had a tetracycline MIC of ≤2 mg l^−1^, while 38 isolates (54.3%) had a MIC > 2 mg l^−1^ and 3 isolates (4.3%) had a vancomycin MIC > 2 mg l^−1^. According to EUCAST guidance [34], a tetracycline and vancomycin MIC > 2 mg l^−1^ suggests that therapy should be discouraged (Table S1).

### Genomic characterization of 20 *L. monocytogenes* isolates from neuroinfections (cerebrospinal fluid and brain abscess samples)

We investigated the genomic diversity of *L. monocytogenes* isolated from the cerebrospinal fluid (*n* = 19) and a brain abscess (*n* = 1) of patients with neuroinfections. The characteristics of the assembled genomes are listed in Table S2.

**Fig. 1.**
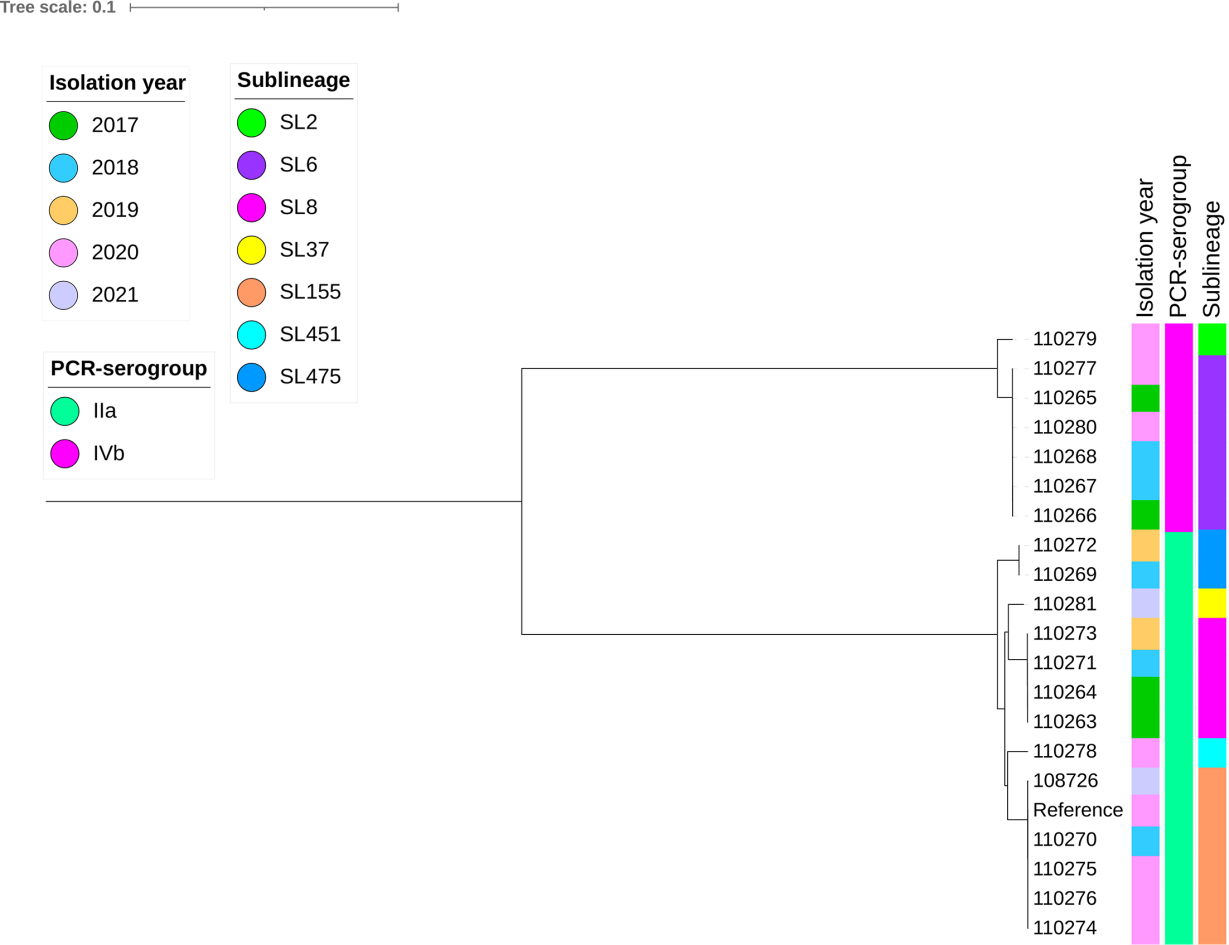
Phylogenetic relationship between 20 *L*. *monocytogenes* isolates from Lithuania. Maximum likelihood phylogenetic tree based on core SNPs of *L. monocytogenes* using the recent ST155 genome (PubMLST assembly ID: 103485_20_06303). Two clades were detected, consistent with the PCR serogroups and sublineages. Five isolates were clustered (6–48 core SNP difference) with the reference genome, isolated during a recent European outbreak [50]. Tree scale indicates nucleotide substitution per site.

Our analysis revealed seven distinct sequence types (STs), seven cgMLST sublineages (SLs) and 16 distinct cgMLST types (CTs) (Table S2). The most common ST was ST6 (*n* = 6), followed by ST155 (*n* = 5), ST8 (*n* = 4) and ST504 (*n* = 2), while ST37, ST451 and ST2 were found in one isolate each (Table S2). Phylogenetic analysis (Fig. 1 and Fig. S3) showed two clades that were separated based on the PCR serogroup: serogroup IVb and IIa isolates were grouped separately. Notably, five isolates were clustered with ST155 (PubMLST assembly ID: 103485_20_06303), which was previously reported in an outbreak in Europe [50].

The isolates differed only by 6–48 core SNPs from the reference and 0, 4 or 44 SNPs from each other, with isolate 110270 from 2018 differing by six SNPs from the reference and only 4 SNPs from isolates 110274, 10276 and 110275. The latter isolates were collected within 2–3 months of each other and differed only by six SNPs different from the reference and 0 SNPs between themselves, suggesting that the ST155 *L. monocytogenes* clone has spread beyond the initial outbreak (the other closest isolate to this clade 110278 was 3665 SNPs different from the reference). However, further investigations would be needed to confirm if these isolates could have been of the reported outbreak or not.

**Fig. 2.**
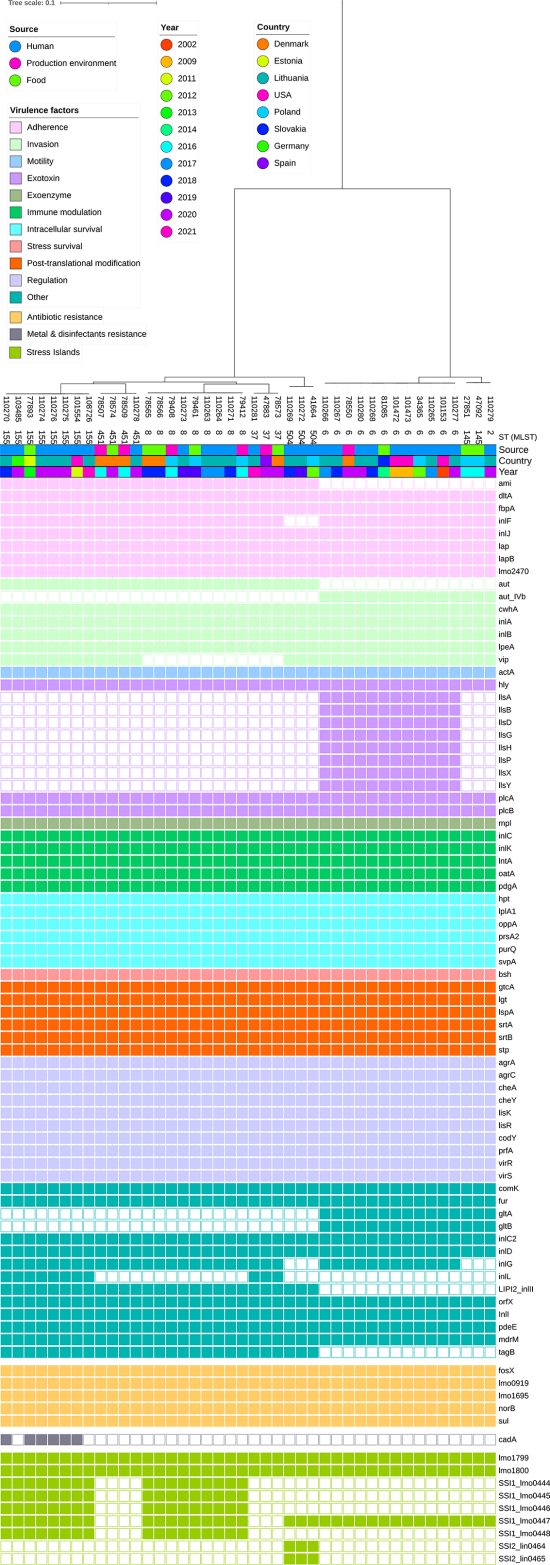
Distribution of virulence, antimicrobial resistance and heavy metal resistance genes among *L. monocytogenes* isolates from Lithuania and genetically related strains from other countries. Maximum likelihood phylogenetic tree of 42 related genomes, including isolates clustered closely with those from Lithuania, based on core SNPs of *L. monocytogenes* using the recent ST155 genome (PubMLST assembly ID: 103485_20_06303). Tree scale indicates nucleotide substitution per site.

Among the relevant virulence genes, stress survival islet 1 (SSI-1), associated with tolerance to acidic, bile, gastric and salt stresses, was detected in 45% (*n* = 9/20) of the isolates, all from lineage II. Seven isolates from lineage I and two from lineage II, harboured only *lmo0447* from SSI-1. SSI-2, associated with survival under high pH and oxidative stresses, was detected in two Lithuanian isolates from lineage II (110269 and 110272). All 20 isolates harboured the *lmo1800* gene, which encodes the phosphatase LipA, essential for promoting infections *in vivo* [54], and *lmo1799*, the putative peptidoglycan-binding protein gene (with a LPXTG motif) (Fig. 2). LPXTG surface proteins play crucial roles in the adhesion and invasion of *L. monocytogenes* [55]. The other annotated genes were functionally categorized into subsystems (Table S4). No genes associated with phosphorus metabolism were detected in any strain.

No acquired antimicrobial resistance genes were detected in any of the 20 genomes; only *fosX* (lmo1702), *lin* (lmo0919), *mprF* (lmo1695), *norB* (lmo2818) and *sul* (lmo0224) were identified, which mediate intrinsic resistance (Table S2, Fig. 2).

### Mobile genetic elements and prophages in *L. monocytogenes*

We detected key plasmids in the assemblies of two isolates, 110263 and 110273, both belonging to ST8. The plasmid in 110263 showed the closest similarity to the plasmid with identifier LR134399 from *L. monocytogenes* strain NCTC7974; this plasmid was 291 717 bp in size, had 300 predicted coding sequences, and a GC content of 37.8% but was identified as a non-mobilizable plasmid through a MOBsuite analysis. The plasmid had genes related to virulence and stress responses (*adhR*, *iap*, *mogR*, *ltaP*, *secA*, *cidC*, *hdeD* and *hflX*), motility and chemotaxis (*fliG*, *fliF*, *fliE*, *fliM*, *fliJ*, *fliN*, *flgC*, *flgB*, *flgE*, *cheA*, *cheV*, *motA* and *motB),* amino acid metabolism and transport and non-coding RNAs (*rli31*, *rli32*, *rli33* and *rli34*) (Table S5).

Isolate 110273 had a plasmid very similar to pLMR479a [56], and was 86 759 bp long, with a GC content of 37.0%, and encoded a Group 2 RepA. The plasmid contained 85 predicted coding sequences (Table S6). The plasmid expressed *cadA1*, *cadA2* and *cadC*, which are associated with resistance to cadmium and other heavy metals [57], as well as *copB* and *copZ*, involved in copper homeostasis and resistance [58,60]. The plasmid also contained the virulence and adaptation genes *virB4*, *traM*, *fetA*, *fetB* and *fixK* and the stress response genes *dps* and *dinB* [61,62].

We identified 11 distinct intact prophages. The most common prophages were *Listeria phage A118* (*n* = 14) and *Listeria phage vB_LmoS_188* (*n* = 11); the rarest were *Listeria phage B054* (*n* = 1) and *Listeria phage LP-030–2* (*n* = 1) (Table S7). All ST155 Lithuanian genomes had an intact *Listeria phage LP-HM00113468*.

### Comparative genomics of Lithuanian and international *L. monocytogenes*

To put our *L. monocytogenes* genomes into a broader geographical context, we compared the Lithuanian *L. monocytogenes* genomes with those from a publicly accessible dataset of *L. monocytogenes* genomes provided by the Institut Pasteur. Using the GrapeTree MST and cgMLST1748 scheme, we identified 297 related isolates (317 including the Lithuanian isolates) (Fig. S1).

**Fig. 3.**
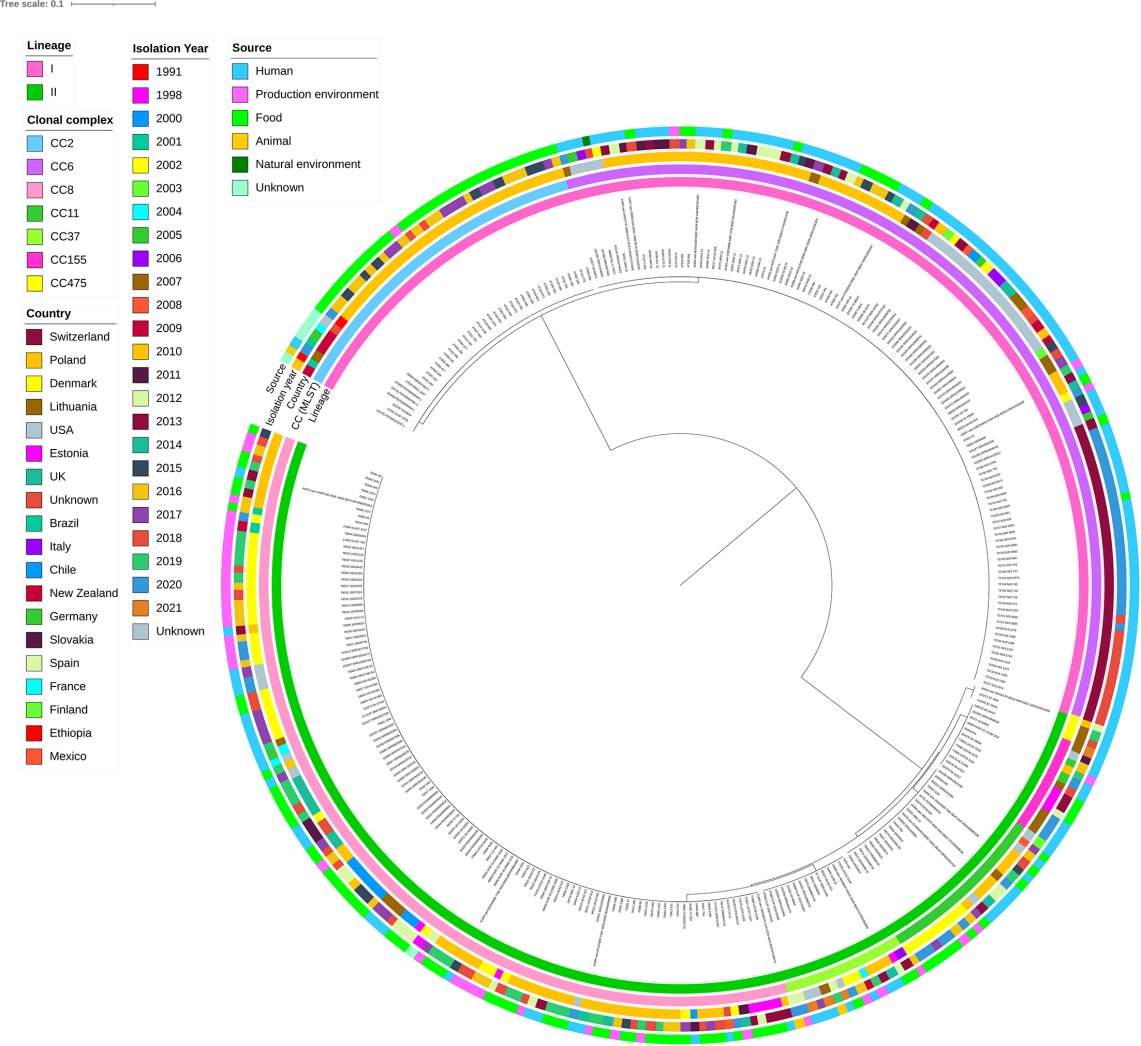
Comparative phylogenetic tree of Lithuanian and other *L. monocytogenes* genomes available from the Institut Pasteur. Maximum likelihood phylogenetic tree of 314 *L*. *monocytogenes* genomes, including 20 genomes from this study, based on core SNPs of *L. monocytogenes* using the recent ST155 genome (PubMLST assembly ID: 103485_20_06303). Tree scale indicates nucleotide substitution per site.

These isolates came from various sources: 150 from clinical samples (47.3%), 115 from food products (36.3%), 42 from food processing environments (13.2%), 3 from animals (0.9%), 1 from the natural environment (0.3%) and 6 with unknown origins, confirming the range of environments *L. monocytogenes* can adapt to. The dataset primarily comprised isolates from Poland (*n* = 126, 39.7%), Denmark (*n* = 41, 12.9%), the USA (*n* = 39, 12.3%) and Lithuania (*n* = 20, 6.3%), with ≤10 isolates collected from other countries (Fig. S1). SNP-based phylogenetic analysis revealed a distinct cluster of isolates that formed clonal complex (CC) 155 (Fig. 3). CC155 contains isolates from humans, as well as food and food production environments; this provides evidence that food-to-human transmission is occurring in these *L. monocytogenes* infections (Fig. 3). The other *L. monocytogenes* isolates were clustered near other isolates from human and food sources (Fig. 3). Overall, *L. monocytogenes* genomes in Lithuania were clustered with genomes from various locations and years.

We compared the distribution of virulence and antimicrobial resistance genes between the Lithuanian and 22 related *L. monocytogenes* genomes (Fig. 2), based on the cgMLST and phylogenetic analyses. Of the 93 virulence genes, 72 (77.4%) distinct genes were detected among isolates (min = 58, max = 67). No significant differences in virulence gene profiles were observed between the isolates from different countries or years, confirming that specific virulence gene profiles are more likely to be associated with * L. monocytogenes* clones than geographical distribution. In total, 30/42 (71.4%) isolates had the *vip* gene, with 22/30 (73.3%) coming from humans (Fig. 2). A total of 12/42 isolates (28.6%) carried the LIPI-3 pathogenicity island, which was most frequently detected in isolates from humans (*n* = 10/27). The adhesion-associated gene *ami*, invasion gene *aut*, LIPI-2 pathogenicity island-associated gene *inlII*, and teichoic acid biosynthesis-related gene *tagB* were found in all serogroup IIa strains but were absent in serogroup IVb strains. Meanwhile, *aut-IVb*, *gltA* and *gltB* and genes within the LIPI-3 pathogenicity island (*llsA*, *llsB*, *llsD*, *llsG*, *llsH*, *llsP*, *llsX* and *llsY*) were only found in serogroup IVb strains, with *aut-IVb*, *gltA* and *gltB* present in all serogroup IVb strains (*n* = 15). All strains carried the LIPI-1 pathogenicity island, which is critical for *L. monocytogenes* infection. Overall, five virulence gene alleles were identified only in Lithuanian strains (Table 1).

**Table 1.** Unique alleles of virulence genes found only in Lithuanian *L. monocytogenes* strains

ID	Locus	Product	Allele
110268	*inlJ*	Internalin J	258
110272	*mpl*	Zinc metalloproteinase precursor	263
110273	*actA*	Actin-assembly inducing protein precursor	608
110277	*actA*	Actin-assembly inducing protein precursor	609
	*inlF*	Internalin F	460

We only identified one gene associated with resistance to heavy metals and disinfectants. The gene *cadA*, which encodes a P-type ATPase and is associated with resistance to heavy metals (cadmium) [63], was found exclusively in six isolates belonging to SL155, including four isolates from Lithuania (110270, 110276, 110275 and 110274), one from the USA (101554) and one from Estonia (77893). We analysed the homologous sequences of the *cadA* gene among the Lithuanian *L. monocytogenes* and other bacteria using BLASTn and identified highly conserved (>99.9%) homologous sequences in *Enterococcus* spp. (e.g. *E. faecium*, *E. faecalis* and *E. saigonensis*).

## Discussion

In recent years, WGS has become the primary tool for epidemiological surveillance in national programmes, outbreak investigations and environmental monitoring in food processing facilities, greatly enhancing food safety controls and public health protection [64,66]. Genomic analysis allows for the rapid classification of bacterial isolates and provides information on the potential phylogenetic relationships. Genomic epidemiology is useful for tracking not only local outbreaks but also those involving multiple institutions and countries [50]. However, in Lithuania, high-resolution methods with strong discriminatory power are not yet implemented in routine listeriosis surveillance. Instead, routine monitoring primarily relies on methods with lower discriminatory capacity, such as serotyping using agglutination reactions or serogroup prediction using PCR-based techniques. In this study, we performed a detailed characterization of 20 *L*. *monocytogenes* isolates from neuroinfection cases using WGS and assessed the antimicrobial susceptibility of clinical isolates collected between 2016 and 2021.

In the EU countries, over 2300 cases of listeriosis are reported annually (the lowest number was observed in 2020, with 1887 cases) [6]. The reported cases in 2023 were the highest in over 5 years, with 2952 cases [6]. The age group most affected was those over 64 years [67]. Notably, Lithuania exhibited a high case fatality rate (average: 24.5% in 2010–2023, peaking at 80% in 2017), contrasting sharply with that of the EU/EEA countries (average: 15.7% in 2010–2023, with a minimum of 13.4% in 2017 and 12.9% in 2020) [68]. However, little is known about the population structure of *L. monocytogenes* from sporadic cases in Lithuania. This study showed that more cases of listeriosis were reported in 2020 and, overall, more cases were detected in patients aged 65 years old and older.

Molecular characterization of *L. monocytogenes* isolates (*n* = 70) from clinical samples in Lithuania identified three serogroups: serogroup IIa and IIc, lineage II (*n* = 54/70); and serogroup IVb, lineage I (*n* = 16/70). This aligns with the findings of previous reports indicating that the majority of human cases are caused by lineages I (serotypes 1/2b and 4b) and II (serotype 1/2 a) [17].

We evaluated the antimicrobial susceptibility of 70 *L*. *monocytogenes* strains using a phenotypic assay based on the standardized broth dilution method, which is considered the gold standard for detecting antimicrobial resistance [69]. All isolates were susceptible to clinically relevant antibiotics, including penicillin G, ampicillin and erythromycin.

WGS analysis of 20 isolates from Lithuania showed that the most common ST was ST6 (*n* = 6), followed by ST155 (*n* = 5), ST8 (*n* = 4), ST504 (*n* = 2), while ST37, ST451 and ST2 were found in one isolate each. Among these, ST6, ST2, ST8, ST37 and ST451 have been associated with invasive listeriosis presenting as meningitis and septicaemia [2,70,73]. In contrast, *L. monocytogenes* genotypes ST8, ST155 and ST504 have been reported as implicated in infection, they have predominantly been isolated from food products, food processing environments and processing water [74,75].

Genomic comparison of the Lithuanian *L. monocytogenes* genomes with similar publicly available genomes showed that the Lithuanian genomes were intermixed with those from other countries, mainly European countries (Poland, Estonia, Denmark, Slovakia, Germany and the USA), isolated from humans, food products or food-processing environments. This is not surprising given the strong relationships reported by an increasing number of molecular epidemiology studies between different sources and geographical areas [50]. Notably, the five Lithuanian ST155 genomes identified in this study were forming a phylogenetic clade with a strain isolated in Germany that belongs to the Omikron1 cluster (ENA code: ERR7113321; PubMLST ID: 103485) and has been associated with an outbreak in Europe [50]; with four isolates <10 core SNPs different and one 48 SNPs different from the ST155 reference used. This Omikron1 strain was classified as serogroup IIa, CC155/SL155 and CT842/CT5098, and the outbreak originated from two food-processing facilities in Lithuania [50]. Omikron1 subcluster 1 strains have been linked to 64 confirmed listeriosis cases from 2016 to 2023 in Austria, Belgium, Germany, Italy and the Netherlands, with 10 fatalities, predominantly occurring in 2020 [50]. Unfortunately, we did not have clinical or detailed metadata on the patients infected with ST155 in this study and could thus not make further extrapolations on these cases and the reported outbreak in Europe.

Notably, we identified an isolate with a plasmid expressing *cadA1*, *cadA2* and *cadC*, which are associated with resistance to cadmium and other heavy metals [57], as well as *copB* and *copZ*, involved in copper homeostasis and resistance [58,60].

*L. monocytogenes* often exhibits tolerance to heavy metals and biocides. The overuse of disinfectants and cadmium resistance may enhance *L. monocytogenes* persistence in food products and food-processing environments [76]. Between 10 and 80% of * L. monocytogenes* strains isolated from food and food processing environments show tolerance to biocides [64]. The persistence of *L. monocytogenes* can elevate the risk of product contamination, food recalls and foodborne outbreaks [77]. The identification of the ST155 outbreak strain from the environment of the two Lithuanian processing plants in 2023 and in products since 2016 reflects the high persistence of *L. monocytogenes* [50] and suggests that the sources of contamination have not been identified and properly controlled.

## Conclusion

The *L. monocytogenes* isolates associated with neuroinfections in Lithuania between 2016 and 2021 were genetically diverse, with five isolates related to the ST155 genotype, which recently caused a multi-country outbreak associated with food production [50]. This indicates that the ST155 genotype was present in the population before the reported outbreak. This retrospective detection underscores the need for a review and strengthening of epidemiological surveillance strategies in Lithuania. Although we did not detect high levels of antimicrobial resistance, the emergence of antimicrobial resistance to first-line treatments, such as aminopenicillins (including amoxicillin, penicillin and ampicillin), has been reported in a few countries [78]. Acquired antimicrobial resistance in *L. monocytogenes* is uncommon, and the standard treatment for listeriosis remains effective. Ongoing surveillance in both clinical and food isolates is essential to identify the emergence of new resistance patterns and evaluate long-term trends in *L. monocytogenes* clone distributions in clinical and non-clinical settings.

## Supplementary material

10.1099/mgen.0.001410 Supplementary Material 1.

10.1099/mgen.0.001410 Supplementary Material 2.

## Abbreviations

CC: clonal complex
EUCAST: European Committee on Antimicrobial Susceptibility Testing
MIC: minimum inhibitory concentration
MST: minimum spanning tree
PD: pharmacodynamic
PK: pharmacokinetic
SL: sublineage
SSI: stress survival islet
ST: sequence type
WGS: whole-genome sequencing
